# Peripheral Immune Alterations in Major Depression: The Role of Subtypes and Pathogenetic Characteristics

**DOI:** 10.3389/fpsyt.2017.00250

**Published:** 2017-11-23

**Authors:** Frank Euteneuer, Katharina Dannehl, Adriana del Rey, Harald Engler, Manfred Schedlowski, Winfried Rief

**Affiliations:** ^1^Division of Clinical Psychology and Psychotherapy, Philipps University of Marburg, Marburg, Germany; ^2^Research Group Immunophysiology, Division of Neurophysiology, Institute of Physiology and Pathophysiology, Philipps University of Marburg, Marburg, Germany; ^3^Institute of Medical Psychology and Behavioral Immunobiology, University Hospital Essen, University of Duisburg-Essen, Essen, Germany; ^4^Department of Clinical Neuroscience, Karolinska Institutet, Stockholm, Sweden

**Keywords:** age of onset, C-reactive protein, cytokines, depressive symptoms, depression subtypes, leukocytes

## Abstract

Depression has been associated with peripheral inflammatory processes and alterations in cellular immunity. Growing evidence suggests that immunological alterations may neither be necessary nor sufficient to induce depression in general, but seem to be associated with specific features. Using baseline data from the Outcome of Psychological Interventions in Depression trial, this exploratory study examines associations between depression subtypes and pathogenetic characteristics (i.e., melancholic vs non-melancholic depression, chronic vs non-chronic depression, age of onset, cognitive-affective and somatic symptom dimensions) with plasma levels of C-reactive protein (CRP), interleukin (IL)-6, IL-10, and numbers of leukocyte subpopulations in 98 patients with major depression (MD) and 30 age and sex-matched controls. Patients with MD exhibited higher CRP levels, higher neutrophil and monocyte counts, lower IL-10 levels, and an increased neutrophil to lymphocyte ratio (NLR) than controls. Patient with later age of onset had higher levels of two inflammatory markers (CRP, NLR) and lower cytotoxic T cell counts after adjusting for sociodemographics, lifestyle factors, and antidepressants. Furthermore, lower anti-inflammatory IL-10 levels were related to more severe somatic depressive symptoms. These results confirm and extend previous findings suggesting that increased levels of CRP are associated with a later onset of depression and demonstrate that also NLR as a subclinical inflammatory marker is related to a later onset of depression.

## Introduction

Depression has been associated with peripheral immune alterations, in particular low-grade inflammation (1, 2). Longitudinal and experimental studies in humans and animals suggest bidirectional immune-to-brain communications, in which inflammation-associated disorders (e.g., cardiovascular disease, metabolic syndrome) and peripheral inflammatory signals lead to depressive symptoms and *vice versa* (3–7).

Importantly, meta-analyses on depression and inflammation suggest a large heterogeneity across studies (1, 2). This heterogeneity may result, in part, from methodological differences and limitations, for example, no consideration of important confounders, inadequate data preparation, or different ways to measure depression. Moreover, heterogeneity may reflect that depression is not a “natural kind” but rather a scientific taxonomic category with substantial within-category variation (8). Confirming this perspective, growing evidence suggests that inflammation may neither be necessary nor sufficient to induce or sustain depression in general, but may be associated with specific subtypes and characteristics of depression, respectively (5). For example, some studies (9–11), but not all (12), suggest that inflammation may be particularly associated with somatic depressive symptoms. Other studies indicate that a later age of onset (13) may relate to higher levels of inflammatory markers, while findings for specific depression subtypes are mixed (14–19).

For immunological measures beside circulating inflammatory markers, results for depression seem to be even more heterogeneous and associations with disease characteristics are understudied. These inconsistencies involve studies on immune cells counts and distributions (20, 21). In addition, results for circulating anti-inflammatory interleukin (IL)-10 are mixed (1, 22, 23), although findings in chronic heart failure (24), renal disease (25), and also animal research (26–31) clearly suggest a link between reduced IL-10 and depression.

Using baseline data from the Outcome of Psychological Interventions in Depression (OPID)-trial (32), this exploratory study examined whether specific depression subtypes [melancholic vs non-melancholic (i.e., atypical or unspecified), chronic vs non-chronic] and characteristics (symptom severity, age of onset) are associated with pro- and anti-inflammatory parameters and leukocyte subsets distribution.

## Materials and Methods

### Participants and Procedure

Data were selected from baseline assessments of the OPID-trial, funded by the German Research Foundation (DFG RI 574/23-1, SCHE 341/20-1) to WR and MS, respectively. As described previously (32), 98 patients aged 18–65 who fulfilled criteria for major depression (MD) in DSM-IV (33) and a sample of 30 age- and sex-matched healthy controls from the same community were included. Patients were recruited *via* the Outpatient Clinic for Psychological Interventions of the University of Marburg, *via* advertisements, leaflets in pharmacies, and waiting rooms of doctors, as well as press releases in local newspapers. Healthy controls were recruited *via* advertisements and press releases in local newspapers. Written informed consent was obtained from all participants.

After prescreening *via* phone, participants underwent a diagnostic session with a clinical psychologist that included the German version of the structured clinical interview for DSM-IV (SCID) (34) and an interview to assess the medical history, potential exclusion criteria, sociodemographics, and several health-related variables. Exclusion criteria were neurological illness, psychotic symptoms, injuries, and infections during the last 14 days, alcohol and/or drug abuse, systemic corticosteroids, antipsychotics, stimulants, current pregnancy and lactation in women, and any mental disorders according to DSM-IV for healthy controls. Because patients with depression were considered for further psychological treatment, they also underwent a physical examination by a physician as requested by German health insurances. Patients who took antidepressants were considered for participation under the assumption that the dose had been stable for at least 2 weeks and would remain so during study participation. After diagnostic sessions and giving informed consent, individuals were invited for blood withdrawal and psychometric measures on the following days.

Pathogenetic characteristics (i.e., melancholic vs non-melancholic depression, chronic vs non-chronic depression, age of onset) were examined during the SCID interview. If available, information from previous doctor visits or hospital reports were also considered. Total depressive symptom severity and scores for cognitive-affective and somatic symptoms (35) were assessed with the German version of the Beck Depression Inventory (BDI)-II (36). The long version of the International Physical Activity Questionnaire (37) was used to assess metabolic equivalent minutes per week (MET-min/week) for total physical activity and three domains (walking, moderate-intensity, and vigorous-intensity).

### Immunological Measures

Before blood sampling, participants were queried about acute infections during the last 14 days, chronic infections, or illness, and a sample was considered missing if participants reported any one of these issues. Participants were instructed to avoid exercise and alcohol 24 h prior to blood withdrawal. Non-fasting blood samples were collected in EDTA-treated or heparinized tubes (S-Monovette, Sarstedt, Nümbrecht, Germany) between 7:00 a.m. and 10:00 a.m. Plasma for C-reactive protein (CRP) and cytokine measurements were separated by centrifugation at 2,000 × *g* for 10 min at 4°C, and plasma was stored at −80°C (7–12 months) until analysis. CRP was measured using an enzyme-linked immunosorbent assay (CRP high-sensitive ELISA, IBL International, Hamburg, Germany) according to the manufacturer’s instructions. Plasma levels of IL-6 and IL-10 were analyzed by flow cytometry using bead-based assays (Bio-Plex Pro Human Cytokine Assays, Bio-Rad Laboratories, Hercules, CA, USA) as previously described (38). The sensitivity of the assays was 0.02 µg/ml for CRP, 0.45 pg/ml for IL-6, and 0.59 pg/ml for IL-10. Undetectable IL-6 (*n* = 1) and IL-10 (*n* = 4) were assigned a value of the limit of detection divided by (LOD/2). Complete blood counts including the white blood cell differential were obtained using an automated hematology analyzer (XT-2000i, Sysmex, Horgen, Switzerland). Leukocyte subpopulations were determined by flow cytometry using a standard lyse/wash procedure and the following antibodies (all from BioLegend, San Diego, CA, USA): FITC-conjugated anti-human CD3 (clone SK7), Pacific Blue-conjugated anti-human CD4 (clone SK3), PE-Cy7-conjugated anti-human CD8 (clone SK1), APC-Cy7-conjugated anti-human CD14 (clone M5E2), PerCP-Cy5.5-conjugated anti-human CD19 (clone HIB19), PE-conjugated anti-human CD25 (clone BC96), PE-conjugated anti-human CD56 (clone MEM-188), and AF647-conjugated anti-human CD127 (clone A019D5). Samples were analyzed on a FACSCanto II flow cytometer (BD Biosciences, Heidelberg, Germany) using BD FACSDiva software (Version 8.0.1, BD Biosciences).

### Statistical Analysis

Statistical analyses were carried out with Mplus7 (Muthén and Muthén, 1998–2012) and IBM SPSS version 23.0 for Windows (Chicago, SPSS, Inc.). Missing values occurred due to heterogeneous technical problems, and extreme outliers in immunological variables were also considered missing values (i.e., values more than three interquartile ranges above the 75th percentile) (39). Immunological data were available as follows: CRP (88%), IL-6 (82%), IL-10 (77%), and leukocyte counts and subsets (83–91%). Differences in group characteristics were calculated using pairwise comparisons with χ^2^ tests and *t*-tests, if necessary with Welch’s correction in case of variance heterogeneity. Associations of depression subtypes and pathogenetic characteristics with immunological measures were analyzed with multiple regression analyses. Associations were examined without adjustment (model 1), with adjustment for sociodemographics (i.e., sex, age; model 2), and with additional adjustment for lifestyle factors (i.e., number of cigarettes/day, body mass index, physical activity; model 3) and antidepressants. *Post hoc* power analysis for linear regression (40) indicated a power of 96.7% with a medium effect size and an alpha of.05. To strengthen robustness and to handle missing data in multivariate analyses, regressions were based on full information maximum likelihood estimation with robust standard errors (MLR). Given the exploratory nature of this study, analyses were not corrected for multiple testing (41).

## Results

### Immunological Alterations in MD

Table 1 shows characteristics of patients with MD and controls. Patients exhibited significantly higher levels of CRP, lower levels of circulating IL-10, as well as higher IL-6/IL-10 ratios, compared to controls. Furthermore, neutrophil counts, monocyte counts, and neutrophil to lymphocyte ratio (NLR) were higher in patients than in controls. There were no significant differences between groups for any other immunological measures.

**Table 1:** Characteristics of patients with MD and controls.

	MD (*n* = 98)	Controls (*n* = 30)	*t* or χ^2^; *p*
Age	37.3 (12.2)	37.1 (12.2)	0.08; 0.939
Female, *n* (%)	48 (49.0)	15 (50.0)	0.01; 0.922
Body mass index, kg/m^2^	26.1 (5.3)	24.0 (4.2)	1.96; 0.052
Education (years)	11.3 (1.7)	12.2 (1.5)	−2.83; 0.006
Number of cigarettes/day	3.0 (7.1)	0.5 (1.9)	1.90; 0.060
Antidepressant medication, *n* (%)			
Total	37 (37.8)	–	–
SSRIs	15 (15.3)	–	–
SNRIs	6 (6.1)	–	–
Agomelatine	5 (5.1)	–	–
NaSSAs	5 (5.1)	–	–
TCAs	5 (5.1)	–	–
St John’s wort	3 (3.1)	–	–
NDRIs	1 (1.0)	–	–
Depressive symptom severity, BDI-II			
Total score	26.9 (9.1)	4.6 (5.5)	18.26; <0.001
Cognitive-affective symptoms	19.3 (7.6)	2.7 (1.9)	15.56; <0.001
Somatic symptoms	7.2 (2.6)	1.9 (1.9)	12.35; <0.001
DSM-IV axis I comorbidity, *n* (%)			
Anxiety disorders	23 (23.5)	–	–
Somatoform disorders	12 (12.2)	–	–
Subtypes, *n* (%)			
Melancholic MD	51 (52.0)	–	–
Chronic MD (≥2 years)	41 (41.8)	–	–
Age of depression onset	28.36 (12.7)	–	–
Physical activity, IPAQ, MET-min/week			
Walking	1,364.8 (1,392.5)	1,919.5 (2,020.6)	−1.61; 0.092
Moderate-intensity activity	2,040.0 (2,227.0)	2,243.8 (2,813.1)	−0.87; 0.388
Vigorous-intensity activity	861.1 (1,670.6)	1,577.3 (1,739.28)	−2.03; 0.045
Total	4.26 (3.56)	5.97 (5.72)	−1.55; 0.130
CRP, μg/ml	1.7 (1.8)	0.8 (0.6)	4.01; <0.001
IL-6, pg/ml	3.9 (5.1)	5.9 (7.8)	−1.22; 0.230
IL-10, pg/ml	5.8 (9.2)	12.9 (16.3)	−2.09; 0.045
IL-6/IL-10 ratio	1.0 (0.9)	0.6 (0.4)	2.71; 0.008
Immune cell counts/μl			
Leukocytes	6,780 (1,889)	6,740 (2,209)	0.69; 0.924
Lymphocytes	2,152 (669)	2,286 (679)	−0.82; 0.413
Neutrophils	4,099 (1,420)	3,520 (989)	2.15; 0.038
Monocytes	403 (134)	310 (153)	2.78; 0.006
Total T cells	1,454 (541)	1,494 (460)	−0.31; 0.761
T helper cells	948 (427)	920 (372)	0.27; 0.789
Cytotoxic T cells	426 (188)	485 (147)	−1.34; 0.184
Regulatory T cells	87 (41)	73 (28)	1.93; 0.061
B cells	236 (147)	197 (94)	1.38; 0.169
NK cells	258 (147)	281 (128)	−0.65; 0.516
Neutrophils/lymphocytes ratio	2.0 (0.7)	1.6 (0.6)	2.50; 0.017

*Values are mean (SD) unless noted with percentage. Group differences were calculated using χ^2^ tests for categorical variables and *t*-tests for continues variables*.

*BDI, Beck Depression Inventory; CRP, C-reactive protein; DSM, Diagnostic and Statistical Manual of Mental Disorders; HC, healthy control group; IL, interleukin; IPAQ, International Physical Activity Questionnaire; MD, major depression; MET, metabolic equivalent; NDRIs, norepinephrine–dopamine reuptake inhibitor; NaSSAs, noradrenergic and specific serotonergic antidepressants; SNRIs, serotonin–norepinephrine reuptake inhibitors; SSRI, selective serotonin reuptake inhibitors; TCAs, tricyclic antidepressants*.

### Association of Depression Subtypes and Pathogenetic Characteristics with Immunological Measures

**Figure 1:**
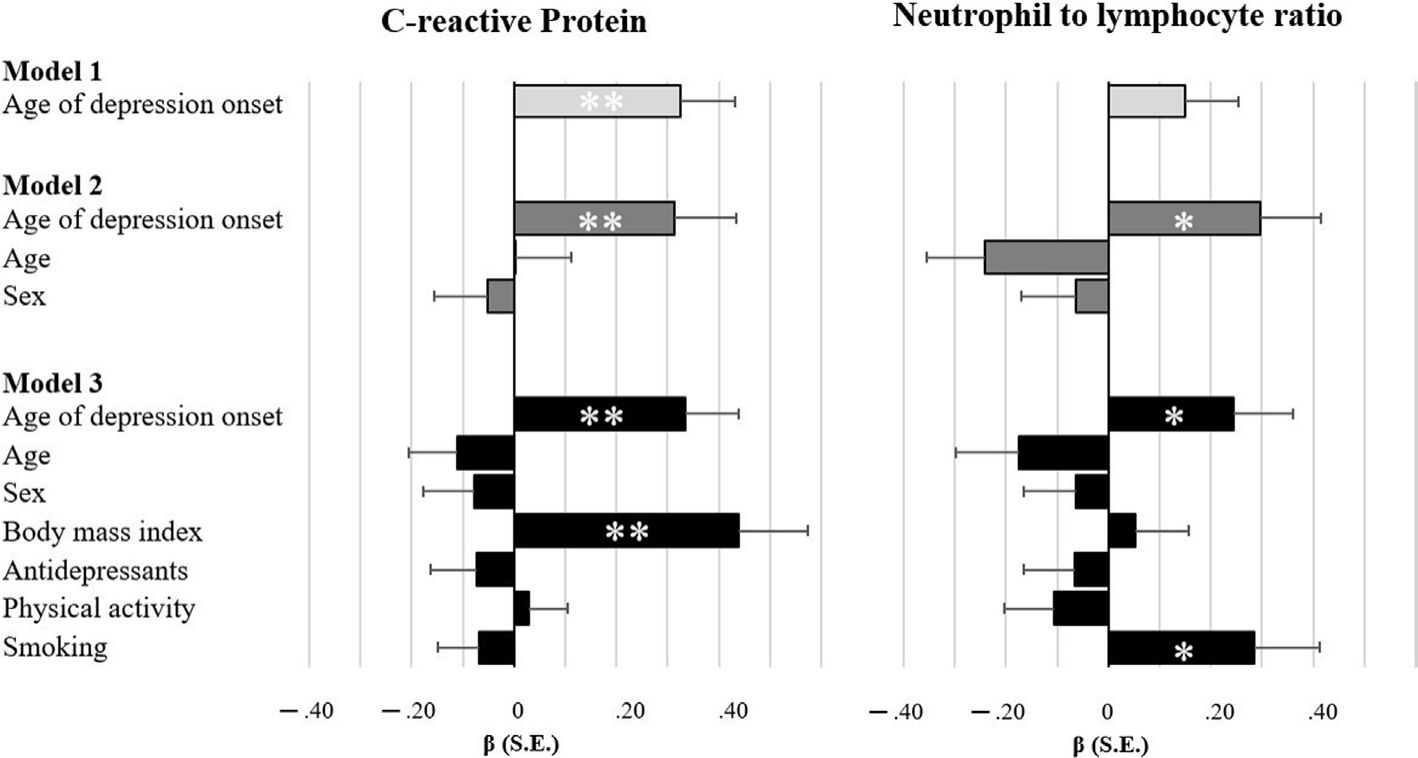
Bar charts illustrating the degree of associations between inflammatory markers and age of depression onset. Standardized estimates based on multiple regressions with full maximum likelihood estimation and robust standard errors. Model 1 is unadjusted, model 2 adjusts for sociodemographics, and model 3 additionally adjusts for lifestyle factors and antidepressant medication. *p < 0.05 **p < 0.01.

As illustrated in Figure 1, age of depression onset was associated with two inflammatory markers. Unadjusted models indicated that patients with later age of onset exhibit higher levels of CRP (β = 0.326, *p* = 0.002). After adjustment for age and sex, patients with later age of onset had higher levels of CRP (β = 0.314, *p* = 0.008) and NLR (β = 0.296, *p* = 0.012). Both associations remained significant, even after full adjustment for lifestyle factors and antidepressants (CRP: β = 0.334, *p* = 0.002; NLR: β = 0.245, *p* = 0.033). Furthermore, although no differences in cytotoxic T cells were observed between patients and controls, cytotoxic T cells were reduced in patients with later age of onset in unadjusted models (β = −0.232, *p* = 0.011) and after controlling for sex and age (β = −0.317, *p* = 0.014), as well as for lifestyle factors and antidepressants (β = −0.305, *p* = 0.025). When looking at associations between immune markers related to age of onset, we found an inverse relationship between NLR and cytotoxic T cells (β = −0.308, *p* < 0.001). CRP was neither significantly associated with NLR (β = −0.087, *p* = 0.403) nor with cytotoxic T cells (β = 0.109, *p* = 0.270).

Finally, we found that patients with higher levels of somatic depressive symptoms had lower levels of IL-10 after adjusting for sex and age (β = −0.209, *p* = 0.017) and additionally adjusting for lifestyle factors and antidepressants (β = −0.238, *p* = 0.038). Immunological measures were not significantly related to any other subtypes or characteristics (see Table S1 in Supplementary Material). Since some previous work on depression and inflammation indicates sex-related association patterns (11, 13, 42), the role of sex was examined in exploratory moderation analyses resulting in no significant interactions.

## Discussion

The aim of this report was to examine whether pro- and anti-inflammatory cytokines and circulating leukocyte subpopulations are associated with specific subtypes and characteristics in MD. As reported and discussed previously (32), patients in this study exhibited higher levels of CRP, lower levels of circulating IL-10, as well as higher IL-6/IL-10 ratios, compared to controls. In addition, neutrophil counts, monocyte counts, and NLR were increased in MD. Our main findings are that patients with later onset of depression exhibit higher levels of two inflammatory markers (i.e., CRP, NLR) and lower levels of cytotoxic T cells after adjusting for sociodemographics, lifestyle factors, and antidepressant medication. We further found that patients with lower levels of anti-inflammatory IL-10 had more severe somatic depressive symptoms.

Our result of an association between CRP and age of depression onset supports previous findings from the Netherlands Study of Depression and Anxiety (NESDA) including a large sample of patients with depression (13). While this study reported that CRP is related to age of onset only in men, we did not observe a moderating role for sex. A simple explanation for this inconsistency might be that women in the NESDA study had a significantly earlier age of depression onset compared to men. In contrast, age of depression onset in our study was comparable between men and women (*p* = 0.764).

In this study, NLR as a marker of subclinical inflammation was increased in patients with MD supporting recent observations (43, 44). Similar to CRP, NLR was related to a later onset of depression. In MD, a later age of onset has not only been associated with inflammation (13), but also with atherosclerosis (45, 46). Also, Kendler et al. (47) found two distinct sets of familial risk factors for MD. While an earlier age of onset was associated with a family history of depression, a later age of onset was associated with a family history of vascular disease. Thus, our findings might be in line with the vascular depression hypothesis as one distinct etiopathological mechanism and support the notion that a later onset of depression is associated with pathological pathways relevant for cardiovascular disease (13).

In this study, lower anti-inflammatory IL-10 was associated with more severe somatic depressive symptom. This finding needs further confirmation, but may be of interest in view of studies demonstrating antinociceptive effects of IL-10 (48) and amplified exhaustion and motor deficits in IL-10-deficient mice after bacterial challenge (49). In contrast to previous work, we did not find evidence for specific associations of inflammatory markers with somatic depressive symptoms (9–11) or depression subtypes (14–19). The reasons for these inconsistencies are unclear, but may relate to differences in study populations and assessment instruments.

This study has important strengths such as the analysis of a wide range of immunological parameters, clinical diagnoses of depression, exclusion of confounding medical conditions, and systematic consideration of potential theoretical confounders. Several limitations should be considered in interpreting our results. First, given the exploratory design of this study, further confirmatory research is necessary. Further limitations are the cross-sectional design and relatively small sample sizes for specific subgroups of patients. Moreover, our sample consisted of outpatients with MD. Thus, findings may not generalize to other samples of patients.

In conclusion, these results confirm and extend previous findings suggesting that increased levels of CRP are associated with a later onset of depression and demonstrate that also NLR as a subclinical inflammatory marker is related to a later onset of depression.

## Ethics Statement

This study was approved by the German Psychological Society Review Board and was in accordance with the International Ethical Guidelines and Declaration of Helsinki. All methods were performed in accordance with the relevant guidelines and regulations.

## Author Contributions

WR and MS were primary investigators. WR, MS, HE, and FE designed and prepared the study. HE and AR prepared and supervised laboratory analyses. KD and FE coordinated data collection. FE wrote the main manuscript text. All authors reviewed the manuscript and contributed to the manuscript text.

## Funding Statement

This research was supported by a grant of the German Research Foundation (DFG RI 574/23-1, SCHE 341/20-1) to WR and MS, respectively.

## Conflict of Interest Statement

The authors declare that the research was conducted in the absence of any commercial or financial relationships that could be construed as a potential conflict of interest.
