# Supplementary material for: Peripheral Immune Alterations in Major Depression: The Role of Subtypes and Pathogenetic Characteristics
Source: Front Psychiatry. 2017 Nov 23;8:250. doi: 10.3389/fpsyt.2017.00250 (PMC5703704; doi:10.3389/fpsyt.2017.00250)
Supplement: Supplementary file 1 [file Table_1.DOCX]

**TABLE S1.**

Associations between immune markers and depression characteristics and subtypes in patients with Major Depression. Comprehensive results for final models adjusted for age, sex, body mass index, antidepressants, physical activity and smoking. Standardized estimates based on multiple regressions with full maximum likelihood estimation and robust standard errors. *p<0.05 **p<0.01 ***p<0.001

|  | Age of depression onset |  | Depressive symptom severity, BDI-II | | |  | Depression subtypes | |
| --- | --- | --- | --- | --- | --- | --- | --- | --- |
|  |  |  | Total | Cognitive-affective | Somatic |  | Melancholic vs non-melancholic | Chronic vs non-chronic |
| CRP, µg/ml  IL-6, pg/ml  IL-10, pg/ml  IL-6/IL-10 ratio  Immune cell counts/µl  Leukocytes  Lymphocytes  Neutrophils  Monocytes  Total T cells  T helper cells  Cytotoxic T cells  Regulatory T cells  B cells  NK cells  Neutrophils/Lymphocytes ratio | .334 (.105)***  -.110 (.140)  .031 (.066)  -.167 (.176)  .078 (.124)  -.111(.137)  .149 (.123)  -.073 (.136)  -.033 (.131)  .046 (.129)  -.305 (.136)*  .106 (.104)  -.230 (.152)  -.218 (.150)  .245 (.115)* |  | .016 (.093)  -.042 (.073)  -.129 (.081)  -.028 (.150)  .131 (.118)  .105 (.095)  .127 (.123)  .050 (.119)  .140 (.087)  .084 (.094)  .005 (.002)  -.002 (.112)  .114 (.100)  -.052 (.090)  .034 (.102) | .049 (.097)  -.056 (.070)  -.061 (.077)  -.100 (.136)  .115 (.093)  .115 (.093)  .084 (.117)  -.036 (.129)  .011 (.001)  .094 (.094)  .007 (.003)  -.001 (.100)  .058 (.104)  -.086 (.091)  -.006 (.089) | .057 (.097)  -.070 (.088)  -.238 (.088)**  .159 (.172)  .051 (.111)  .001 (.095)  .068 (.123)  -.069 (.120)  .011 (.001)  .004 (.015)  .006 (.008)  -.034 (.100)  .027 (.101)  -.042 (.109)  .084 (.113) |  | -.044 (.107)  .000 (.111)  -.167 (.104)  .044(.103)  -.052 (.108)  -.030 (.112)  -.065 (.104)  -.049 (.108)  -.067 (.109)  .017 (.107)  .079 (.115)  .054 (.110)  -086 (.107)  -.001 (.111)  -.001 (.102) | .177 (.102)  .029 (.117)  .065 (.106)  -.038 (.140)  -.070(.100)  -.001 (.109)  -.128 (.099)  .226 (.111)  .017 (.106)  .033 (.094)  -.043 (.111)  . 010 (.096)  -.024 (.121)  -.077 (.110)  -.130 (.108) |

Values are standardized estimates (standard errors). BDI, Beck Depression Inventory; CRP, C-reactive protein; IL, Interleukin.
